# How Compliance of Surfaces Affects Ankle Moment and Stiffness Regulation During Walking

**DOI:** 10.3389/fbioe.2021.726051

**Published:** 2021-10-05

**Authors:** Kaifan Xie, Yueling Lyu, Xianyi Zhang, Rong Song

**Affiliations:** ^1^ Key Laboratory of Sensing Technology and Biomedical Instrument of Guangdong Province, Sun Yat-sen University, Guangzhou, China; ^2^ Guangdong Provincial Engineering and Technology Center of Advanced and Portable Medical Devices, Sun Yat-sen University, Guangzhou, China

**Keywords:** ankle biomechanics regulations, EMG-driven musculoskeletal model, muscle excitations, compliant surfaces, gait analysis

## Abstract

Humans can regulate ankle moment and stiffness to cope with various surfaces during walking, while the effect of surfaces compliance on ankle moment and stiffness regulations remains unclear. In order to find the underlying mechanism, ten healthy subjects were recruited to walk across surfaces with different levels of compliance. Electromyography (EMG), ground reaction forces (GRFs), and three-dimensional reflective marker trajectories were recorded synchronously. Ankle moment and stiffness were estimated using an EMG-driven musculoskeletal model. Our results showed that the compliance of surfaces can affect both ankle moment and stiffness regulations during walking. When the compliance of surfaces increased, the ankle moment increased to prevent lower limb collapse and the ankle stiffness increased to maintain stability during the mid-stance phase of gait. Our work improved the understanding of gait biomechanics and might be instructive to sports surface design and passive multibody model development.

## Introduction

The ankle joint plays several important roles such as shock absorption, stability, and propulsion in different subphases of the gait cycle, which can be realized by the regulations of ankle biomechanics (Robertson and Winter, 1980; Neptune et al., 2001). When environments change, the ankle joint adapts its biomechanical properties accordingly (Winter, 1995; Bayram and Bayram, 2018). These adaptions include the regulations of ankle moment and stiffness (Kepple et al., 1997; Whitmore et al., 2019). Ankle moment and stiffness are both regulated primarily by ankle muscles and can be regulated at different levels by co-activation of the agonist and antagonist muscles. For instance, enhanced activation of agonist and antagonist can increase the ankle stiffness while keeping the net ankle moment constant (Lee et al., 2014; Wind and Rouse, 2020).

In order to learn about the ankle moment and stiffness regulations, several methods have been developed to estimate ankle moment and stiffness. Ankle moment can be estimated from muscle forces and their associated moment arms (Sartori et al., 2012; Sartori et al., 2014). An inverse dynamics approach has been also used to estimate ankle moment by solving for the unknowns in the algebraic equations which take segmental anthropometry, lever arms, and movements measured as input (Vaughan and Christopher, 1996). The main sources of error in this approach are the inaccuracy in movement coordination data and estimations of body segment parameters (Riemer and Hsiao-Wecksler, 2008). Ankle stiffness is an important component of ankle impedance and can be estimated from the isolated angle and torque response to the perturbation applied to the ankle joint (Rouse et al., 2014; Lee and Hogan, 2015; Shorter and Rouse, 2018). In recent years, some perturbing robots have been developed to apply perturbations to the ankle joint in a certain period of the gait cycle. A majority of previous studies obtained joint stiffness from the slope of the joint moment–angle curve directly (Gunther and Blickhan, 2002; Yoon et al., 2007; Mager et al., 2018), which is referred to as quasi-stiffness (Latash and Zatsiorsky, 1993). However, due to the positive work produced by muscles during joint movements, quasi-stiffness is not a reasonable representation of joint stiffness (Rouse et al., 2013). An alternative way is to derive continuous ankle stiffness from the stiffness of constituent muscle–tendon units (MTU) using an EMG-driven musculoskeletal model (Sartori et al., 2015).

Previous studies demonstrated that ankle moment and stiffness were regulated according to the subphase of the gait cycle and walking environments. During the early stance phase, the ankle dorsiflexion moment is generated to provide preparation for weight acceptance. During the mid- and late stance phases, the ankle plantar flexion moment generated contributes to support and forward progression (Kepple et al., 1997; Sadeghi et al., 2001). The ankle stiffness increases from heel strike, reaching maximum in the late stance phase and then decreases to a low value before toe-off (Lee et al., 2016). This regulation of ankle stiffness matches with the need to prevent foot slap following heel strike and maintains stability during the stance phase (Lee et al., 2016). When the walking environment changes, the ankle moment and stiffness can be regulated to cope with the change (Ferris et al., 1998; Ferris et al., 1999; Yang and Pai, 2010; Whitmore et al., 2019; Yoo et al., 2019). For example, during the first exposure to a novel and unannounced slippery surfaces, ankle plantar flexion moments would reduce during the late stance phase for slippery recovery (Yang and Pai, 2010). When the swing limb was tripped by surface obstacles, larger ankle plantar flexion moment was generated on the supporting limb to provide adequate time and clearance for positioning of the recovery foot (Pijnappels et al., 2005). A recent study found that ankle stiffness decreased in the late stance phase while walking on a slippery surface to avoid falls (Whitmore et al., 2019). It has been shown that leg stiffness increased during the stance phase while running on more compliant surfaces, which may improve body stability on compliant surfaces (Ferris et al., 1998; Ferris et al., 1999). As leg stiffness primarily depends on the ankle joint stiffness (Farley and Morgenroth, 1999), increased leg stiffness on compliant surfaces may primarily result from the ankle stiffness.

Although many factors can affect the ankle moment and stiffness, it remains unclear how humans regulate them while walking on surfaces with different levels of compliance. Our study aimed to determine how humans regulate ankle moment and stiffness while walking on surfaces with different levels of compliance. As each subphase of gait has distinct biomechanical demands, we hypothesized that the compliance of surfaces may affect ankle moment and stiffness regulations in different subphases of a gait cycle. An EMG-driven musculoskeletal model was applied to estimate ankle moment and stiffness while walking on surfaces with different levels of compliance.

## Materials and Methods

### Materials

Three materials were selected to form three surfaces for subjects to walk on. The materials selected were rubber, ethylene–vinyl acetate copolymer (EVA), and expandable polyethylene (EPE). The surface of the force plate made up of aluminum was another surface for subjects to walk on and its elastic modulus was about 70,000 MPa. A universal material testing machine was used to obtain the force deformation data of the samples of the materials. The thickness of the samples was 5 cm. Then the elastic modulus of materials was obtained from the relationship of stress and strain (0–300 kPa). The relationship of stress and strain is shown in Supplementary Figure 1. The elastic modulus of rubber, EVA, and EPE was 4.10, 0.34, and 0.29 MPa, respectively.

### Experiment Setup

Subjects were required to walk barefoot during the experiment. Before starting walking trials on one surface, subjects were required to walk on this surface to adapt to it. One static pose trial was performed before walking trials. Then subjects were required to perform four walking trials on each surface. In walking trials, subjects were required to walk across a walkway of a length of 4.5 m in 4–5 s. The subjects were allowed to take a 1-min rest between each pair of successive walking trials and a 15-min rest when the surfaces for walking trials needed to be changed. One subject was recruited for the pilot study, and the ankle stiffness estimates were obtained using an EMG-driven musculoskeletal model. Effect sizes [Cohen’s *f* (Cohen, 1969)] of the ankle stiffness estimated during mid- and late stance phases in the pilot study were greater than those obtained using 1.5. Software G*power used for sample size calculation. As a result, the sample size required was eight for repeated measures ANOVA with an effect size *f* value of 1.5, an α value of 0.05, and a power value of 0.8. Then ten healthy subjects (male, 63.46 ± 7.73 kg, 23.20 ± 1.54 years old) without lower extremity injury participated in the experiment. All subjects signed the informed consent form before participating in the experiment. This study was approved by the School of Medicine, Sun Yat-sen University Institutional Review Board, on March 1, 2021.

Recordings of walking trials included the whole stance phase of the subjects’ right leg. EMG data were collected from four ankle muscles: tibialis anterior (TA), soleus (SOL), gastrocnemius lateralis (GAL), and gastrocnemius medialis (GAM). EMG data were recorded at 1,500 Hz using a telemetered EMG system (Noraxon, Scottsdale, USA). Electrodes were placed on these muscles using surface EMG for non-invasive assessment of muscles (SENIAM) guidelines. (Details are available at http://www.seniam.org/) GRF data were recorded at 1,500 Hz using a force plate (Kistler, Winterthur, Switzerland). Each subject had 30 retroreflective markers placed on their body during the experiment. The retroreflective markers were placed on the trunk, pelvis, and left and right extremities. The placements of retroreflective markers are shown in Supplementary Figure 2. Marker trajectories were recorded at 100 Hz using a 6-camera motion capture system (Motion Analysis Corporation, Santa Rosa, USA). EMG, GRFs, and marker trajectories were all collected synchronously.

### EMG-Driven Musculoskeletal Model

**FIGURE 1:**
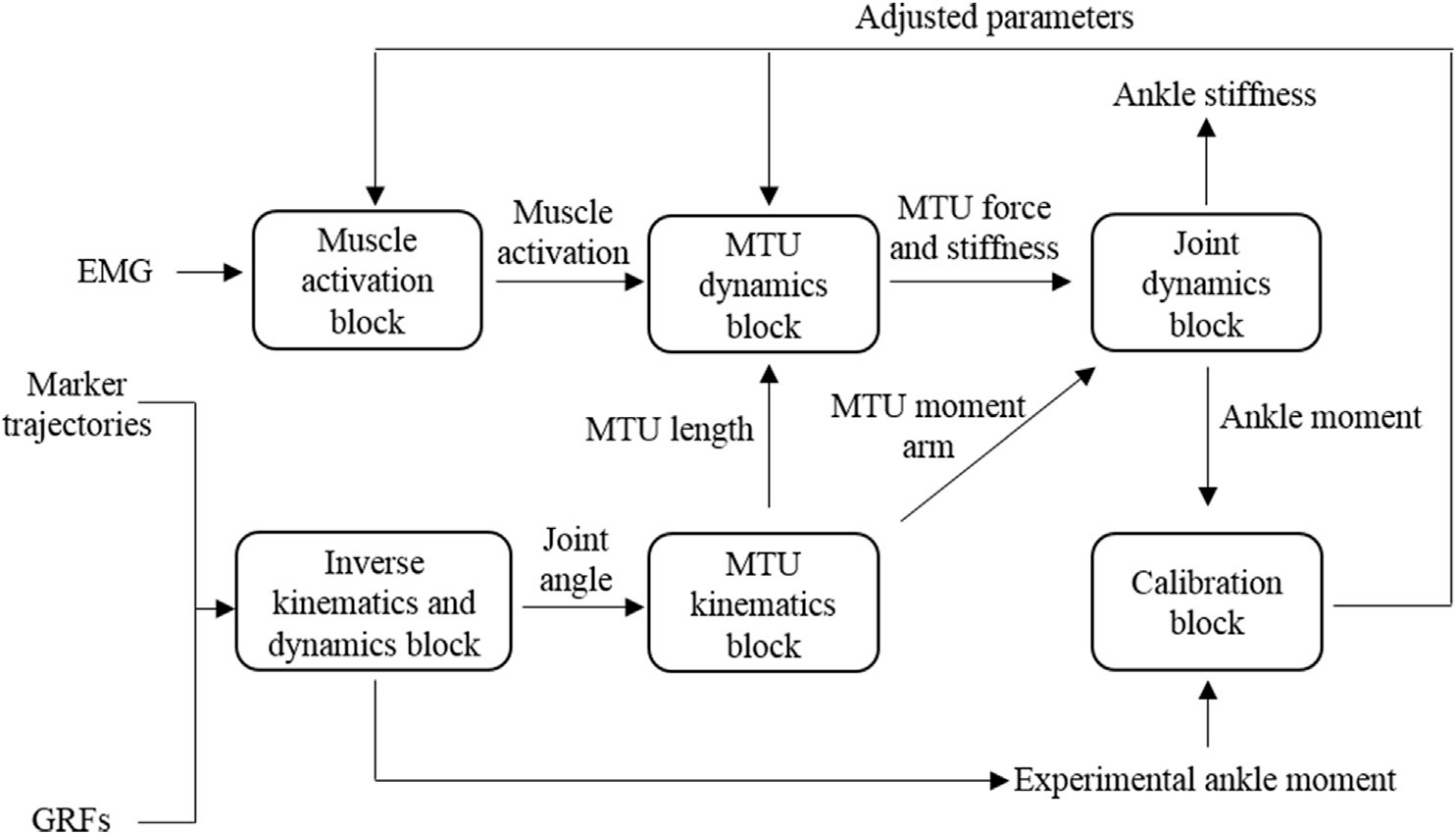
Schematic structure of electromyography (EMG)-driven musculoskeletal model.

The schematic structure of an EMG-driven musculoskeletal model for ankle moment and stiffness estimation is shown in Figure 1, similar to the model proposed by Sartori et al. (2015) *.* The EMG-driven musculoskeletal model includes six blocks.

In the muscle activation block, raw EMG data were band-pass–filtered (30–450 Hz), full-wave–rectified, and low-pass–filtered (6 Hz) using a zero-phase second-order Butterworth filter. For each subject and muscle, the resulting EMG linear envelopes were normalized to the maximum processed values obtained from all recorded trials. The processed and normalized EMG signals would be referred to as excitations. Muscle excitations were subsequently processed using a recursive filter to model the twitch response of the muscle fibers to the excitation onset. The filter used was given by Lloyd and Besier (2003):

$$\begin{array}{c} u\left(t\right)=\alpha e\left(t-d\right)-\beta_{1}u\left(t-1\right)-\beta_{2}u\left(t-2\right) \end{array},$$ (1)

where u(t) is the neural activation and *d* is the electromechanical delay, and a set of constrains were employed given as follows:

$$\begin{array}{c} \{\begin{array}{c} \beta_{1}=C1+C2\\ \beta_{2}=C1\cdot C2\\ \alpha -\beta_{1}-\beta_{2}=1.0 \end{array} \end{array},$$ (2)

where |C1| <1 and |C2| <2. The values of *C1* and *C2* changed the impulse response of the filter.

Then a non-linear transfer function was used to account for the non-linearity in the excitation-to-force relationship and obtain the resulting muscle activation (Lloyd and Besier, 2003):

$$\begin{array}{c} a\left(t\right)=\frac{e^{Au\left(t\right)}-1}{e^{A}-1} \end{array},$$ (3)

where a(t) is the muscle activation and *A* is the non-linear shape factor.

In the inverse kinematics and dynamics block, marker positions recorded from the static pose trials were used to scale a generic model of the human musculoskeletal geometry to match each subject’s anthropometry in OpenSim. Joint angles were calculated using marker trajectories from walking trials *via* the inverse kinematics (IK) tool. Ground reaction forces (GRFs) and the results of IK were then used to calculate ankle moment $\tau_{ID}$ *via* the inverse dynamics (ID) tool. Ankle moment obtained by this way would be referred to as experimental ankle moment.

The MTU kinematics block received joint angles from the inverse kinematics tool in OpenSim. The MTU lengths and moment arms derived from the scaled model in OpenSim were used to create polynomial fitting functions. These functions described how each MTU length and moment arm change with respect to joint angles (Menegaldo et al., 2004). With these polynomial fitting functions and IK-generated joint angles, time-varying MTU lengths and moment arms in walking trials could be obtained.

The MTU dynamics block took muscle activation and MTU lengths from previous blocks as input. A hill-type muscle model was used to estimate the instantaneous muscle fiber length and force and series elastic tendon strain and force for each MTU (Hill et al., 1938; Zajac, 1989; Hoy et al., 1990):

$$\begin{array}{c} F_{m}=F_{max}\left[f\left({\tilde{l}}_{m}\right)f\left({\tilde{v}}_{m}\right)a+f_{P}\left({\tilde{l}}_{m}\right)\right] \end{array},$$ (4)

$$\begin{array}{c} F_{t}=\{\begin{array}{c} 0,\;\;\;\;\;\;\;\;\;\;\;\;\;\;\;\;\;\;\;\;\;\;\;\;\varepsilon \leq 0\\ 1480.3F_{max}\varepsilon^{2},\;\;\;\;\;\;\;0<\varepsilon \leq 0.0127\\ \left(37.5\varepsilon -0.2375\right)F_{max},\;\;\varepsilon \geq 0.0127 \end{array} \end{array},$$ (5)

$$\begin{array}{c} \varepsilon =\frac{l_{t}-l_{st}}{l_{st}} \end{array},$$ (6)

$$\begin{array}{c} F_{mt}=F_{t}=F_{m}cos\Phi \end{array},$$ (7)

where $F_{m}$ is the fiber force; $F_{t}$ is the tendon force; $F_{mt}$ is the MTU force; $F_{max}$ is the maximum isometric muscle force; $f\left({\tilde{l}}_{m}\right)\;$ and $\;f\left({\tilde{v}}_{m}\right)$ are the active force–length relationship (Giat et al., 1994) and force–velocity relationship (Schutte and Rodgers, 1993), respectively; ${\tilde{l}}_{m}$ is the fiber length normalized to the optimal fiber length $l_{m0}$ ; ${\tilde{v}}_{m}$ is the ratio of current muscle fiber velocity to the maximum contraction velocity; a is the muscle activation; $f_{P}\left({\tilde{l}}_{m}\right)$ is the passive elastic force–length relationship (Buchanan et al., 2004); $l_{t}$ is the current tendon length; $l_{st}$ is its slack length; and Φ is the pennation angle.

Muscle fiber stiffness $K_{m}$ is calculated as the partial derivative of fiber force $F_{m}$ with respect to the fiber length $l_{m}$ :

$$\begin{array}{c} K_{m}=\frac{\partial \;F_{m}}{\partial \;l_{m}} \end{array}.$$ (8)

$K_{t}$ is defined by the slope of the force–strain curve of tendon as follows:

$$\begin{array}{c} K_{t}=\frac{d\;F_{t}}{d\;l_{t}} \end{array}.$$ (9)

The MTU stiffness could be modeled as the muscle fiber stiffness $K_{m}$ in series with the tendon stiffness $K_{t}$ as follows:

$$\begin{array}{c} K_{mt}={\left(\frac{1}{K_{m}}+\frac{1}{K_{t}}\right)}^{-1} \end{array}.$$ (10)

The joint dynamics block computed ankle moment and stiffness. Ankle moments were calculated as the product of each MTU force and their associated moment arms, as follows:

$$\begin{array}{c} \tau_{EMG}=\sum_{i=1}^{\#MTU}F_{mt_{i}}\cdot r_{i} \end{array},$$ (11)

where $\tau_{EMG}$ is the ankle moment, $F_{mt_{i}}$ is the force of the *i*th MTU, and $r_{i}$ is the moment arm with respect to the ankle joint of *i*th MTU.

Using the estimated muscle forces and the MTU stiffness, the corresponding ankle stiffness ${\text{K}}_{ankle}$ was computed as follows (McIntyre et al., 1996):

$$\begin{array}{c} K_{ankle}=\sum_{i=1}^{\#MTU}K_{mt_{i}}\cdot r_{i}^{2}+\frac{\partial r_{i}}{\partial \theta }\cdot F_{mt_{i}} \end{array},$$ (12)

where $K_{mt_{i}}$ is the stiffness of the *i*th MTU, $r_{i}$ is the moment arm with respect to the ankle joint of *i*th MTU, θ is the ankle joint angle, and $F_{mt_{i}}$ is the force of the *i*th MTU.

The calibration block determined subject-specific parameters for the EMG-driven musculoskeletal model. Some parameters were adjusted through the calibration process within moderate bounds so that joint moments calculated from MTU forces and moment arms in joint dynamics block could be closer to the joint moments calculated *via* ID in the inverse kinematics and dynamics block. The optimization formulation is listed as follows:

$$\begin{array}{c} J=\frac{1}{N}\sum_{i=1}^{N}{\left(\tau_{EMG}\left(i\right)-\tau_{ID}\left(i\right)\right)}^{2} \end{array},$$ (13)

where *N* represented the length of the data used for the calibration. The Nelder–Mead algorithm was used to minimize the objective function J . The input parameters of the calibration block and their bounds are as follows: *C1* and *C2* in the muscle activation block, which varied between −1 and 1; *A* in the muscle activation block, which varied between −3 and 0; $F_{max}$ in the MTU dynamics block were adjusted by strength coefficients $\gamma_{1}$ and $\gamma_{2}$ for ankle dorsi flexors and ankle plantar flexors, respectively, and $\gamma_{1}$ $\gamma_{2}$ varied between 0.5 and 1.5; and in the MTU dynamics block, the optimal muscle fiber length was adjusted so that $l_{m0}=\text{initial value}\;\pm 2.5\%$ and the tendon slack length were adjusted so that $l_{st}=\text{initial value}\;\pm 5\%$ . The initial $F_{max}$ , $\;l_{m0}$ , and $l_{st}$ were obtained from the scaled model in OpenSim. The calibration process was conducted because some parameters of MTU were different among individuals. Using a calibration process, the parameters could be adjusted to individual values.

### Data Analysis

Walking speeds were calculated from the marker placed on the seventh cervical vertebra of subjects. The ankle moment and stiffness were estimated from the recorded GRFs, EMG, and marker trajectories *via* the EMG-driven musculoskeletal model. Similarity between $\tau_{EMG}$ in the joint dynamics block and the experimental ankle moment in the inverse kinematics and dynamics block after calibration was calculated using the root mean squared error normalized with respect to the root mean squared sum of the corresponding experimental ankle moment (NRMSE) of each subject.

The presence of significant differences among surfaces in ankle moment, ankle stiffness, GRFs, and muscle excitations was assessed with 1D statistical parametric mapping (SPM). SPM represented the convergence of change distribution analysis and significance probability mapping (Friston et al., 1994). One-way repeated measure ANOVA of 1D SPM was performed using an open-source code in MATLAB (MatlabR 2014a, MathWorks Inc., Natick, USA). Details of the SPM analysis and the code are available at https://spm1d.org/.

## Result

The mean (standard deviation) speeds of walking trials on force plate, rubber, EVA, and EPE surfaces were 0.90 (0.06), 0.90 (0.05), 0.88 (0.05), and 0.89 (0.05) m/s, respectively. The mean (standard deviation) NRMSE of ten subjects showing similarity between $\tau_{EMG}\;$ and experimental ankle moment was 0.353 (0.043).

**FIGURE 2:**
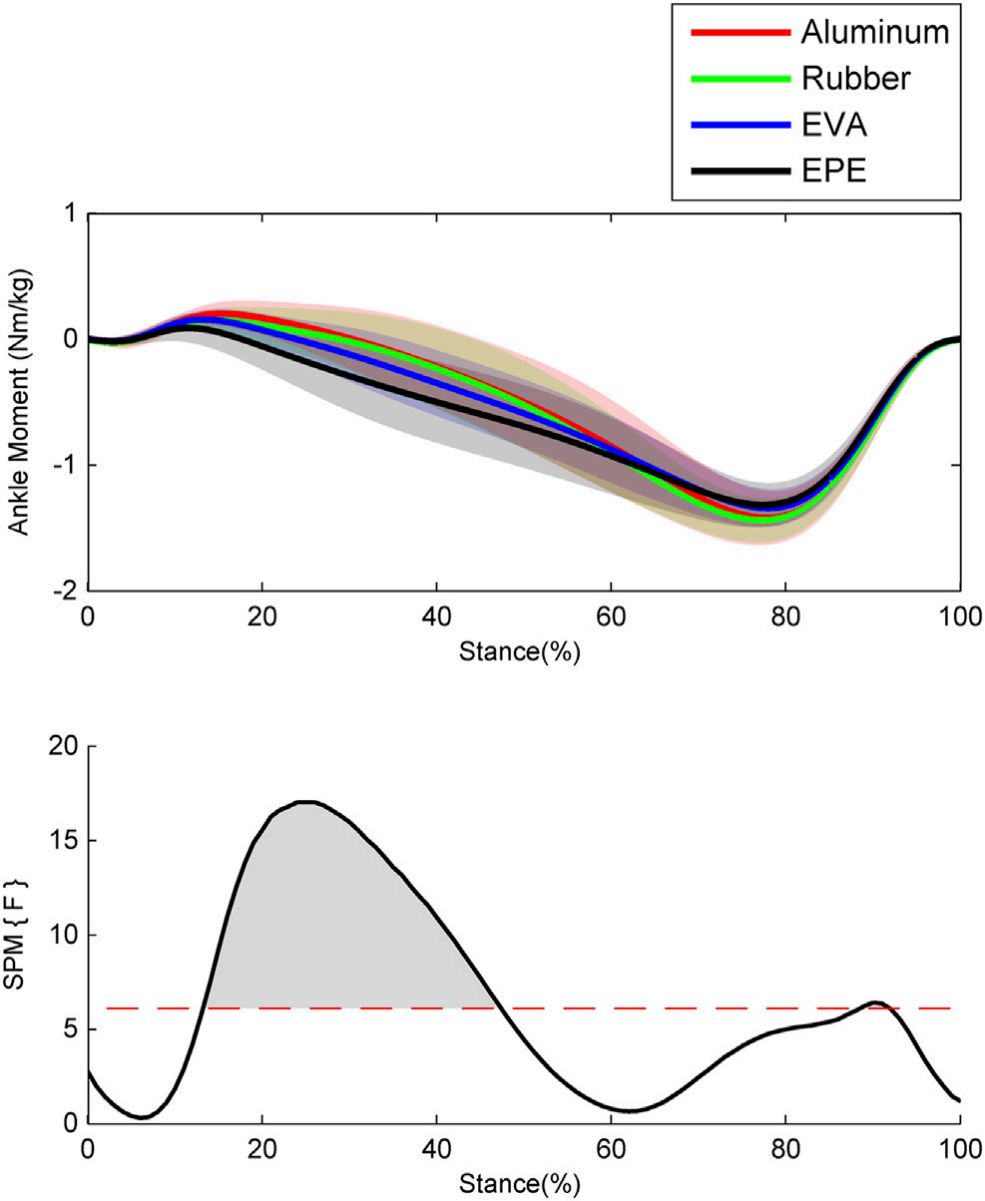
Ensemble average curves (continuous line) and standard deviation (shaded area) for ankle moment during walking. The positive values indicate dorsiflexion moment, and the negative values indicate plantar flexion moment. Data are reported for the stance phase of walking with 0% being heel strike and 100% being toe-off events. The shaded areas in the SPM figure indicate that a significant difference was found (*p* < 0.05) during this period.

The ankle moments calculated *via* ID in OpenSim are shown in Figure 2. During the early stance phase around the 0–20% of stance phase, ankle moments on surfaces with higher compliance were smaller and changed from dorsiflexion to plantar flexion earlier. During the 30–47% of stance phase where ankle moments on all four surfaces have changed into plantar flexion, ankle moments increased as compliance of walking surfaces increased within the force plate, EVA, and EPE surfaces (*p* < 0.001, effect size *f*: 0.27–0.48). During the late stance phase, almost no significant difference existed in ankle moments. During the whole stance phase, ankle moments remained almost the same while walking on the force plate and rubber surfaces.

**FIGURE 3:**
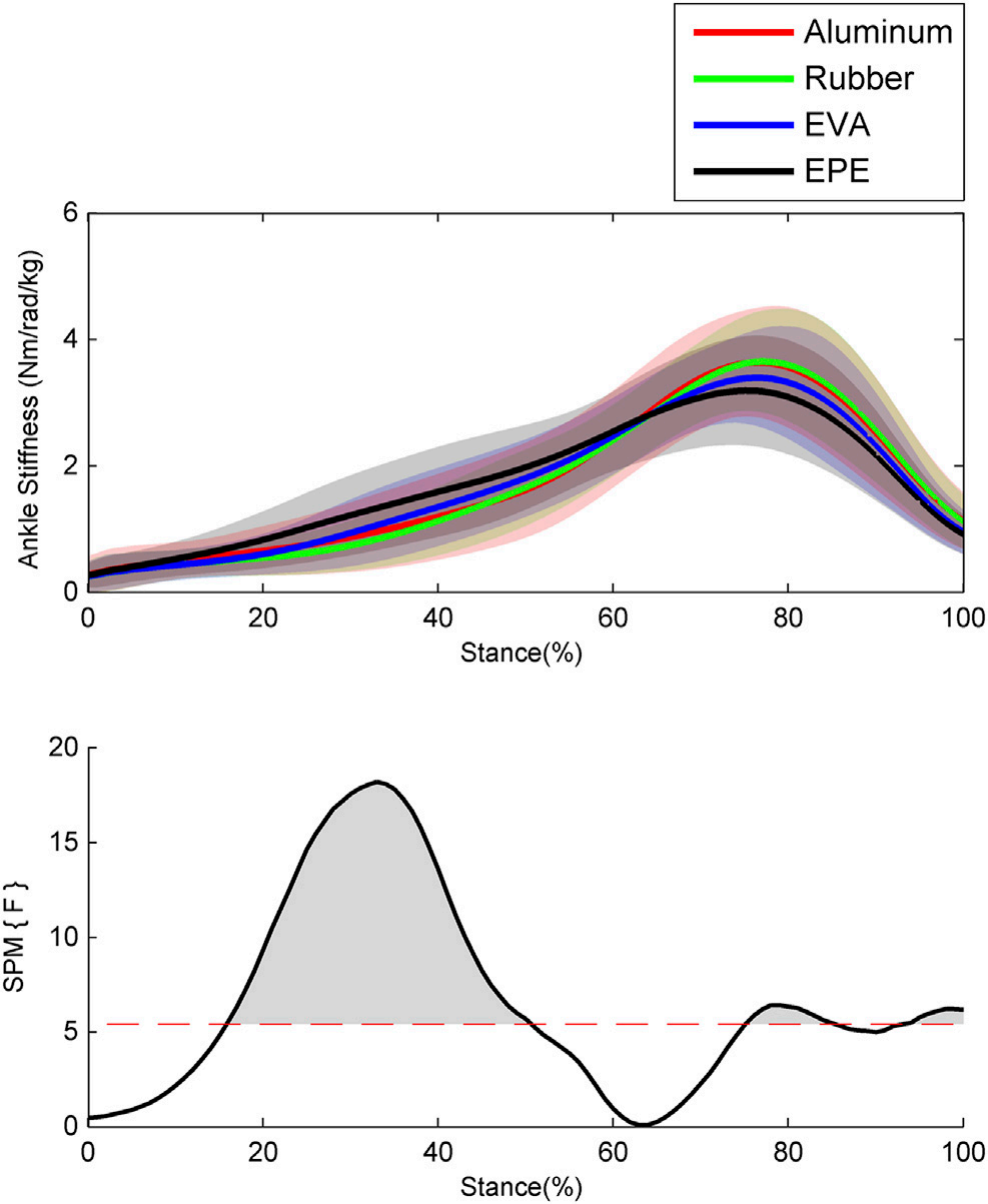
Ensemble average curves (continuous line) and standard deviation (shaded area) for ankle stiffness during walking. Data are reported for the stance phase of walking with 0% being heel strike and 100% being toe-off events. The shaded areas in the SPM figure indicate that significant difference was found (*p* < 0.05) during this period.

Results of the ankle stiffness are shown in Figure 3. During the 40–50% of stance phase, the ankle stiffness increased as compliance of surfaces increased within the force plate, EVA, and EPE surfaces (*p* < 0.001, effect size *f*: 0.22–0.28), while this trend reversed during the 76–85% (*p* = 0.023, effect size *f*: 0.20–0.23) and 94–100% (*p* = 0.035, effect size *f*: 0.21–0.25) of stance phase. Similar to the pattern of ankle moment, the ankle stiffness on the force plate and rubber surface remained almost the same during the whole stance phase.

**FIGURE 4:**
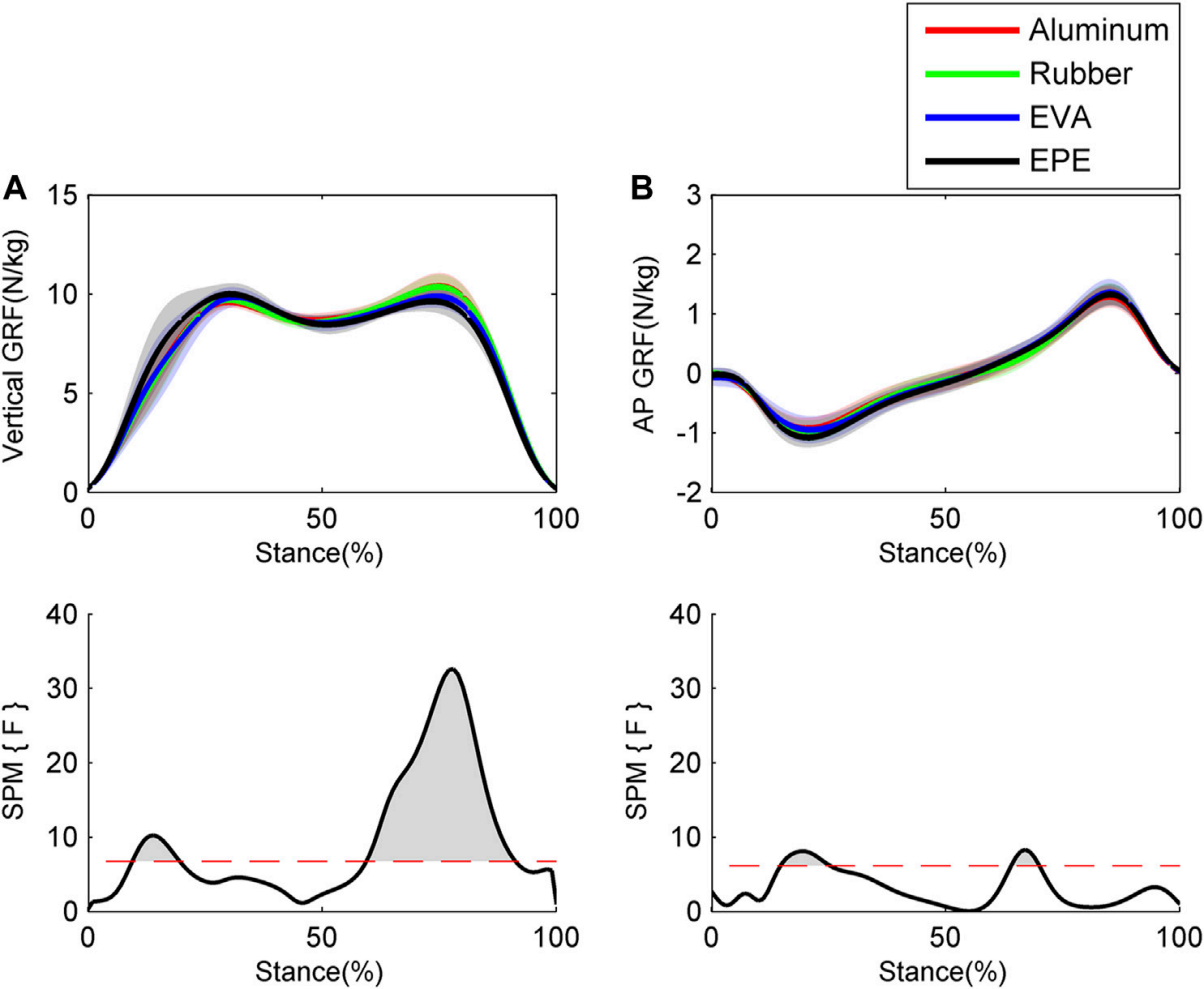
Ensemble average curves (continuous line) and standard deviation (shaded area) for GRFs during walking: **(A)** vertical GRF; **(B)** anterior–posterior (AP) GRF. Data are reported for the stance phase of walking with 0% being heel strike and 100% being toe-off events. The shaded areas in the SPM figure indicate that significant difference was found (*p* < 0.05) during this period.

**FIGURE 5:**
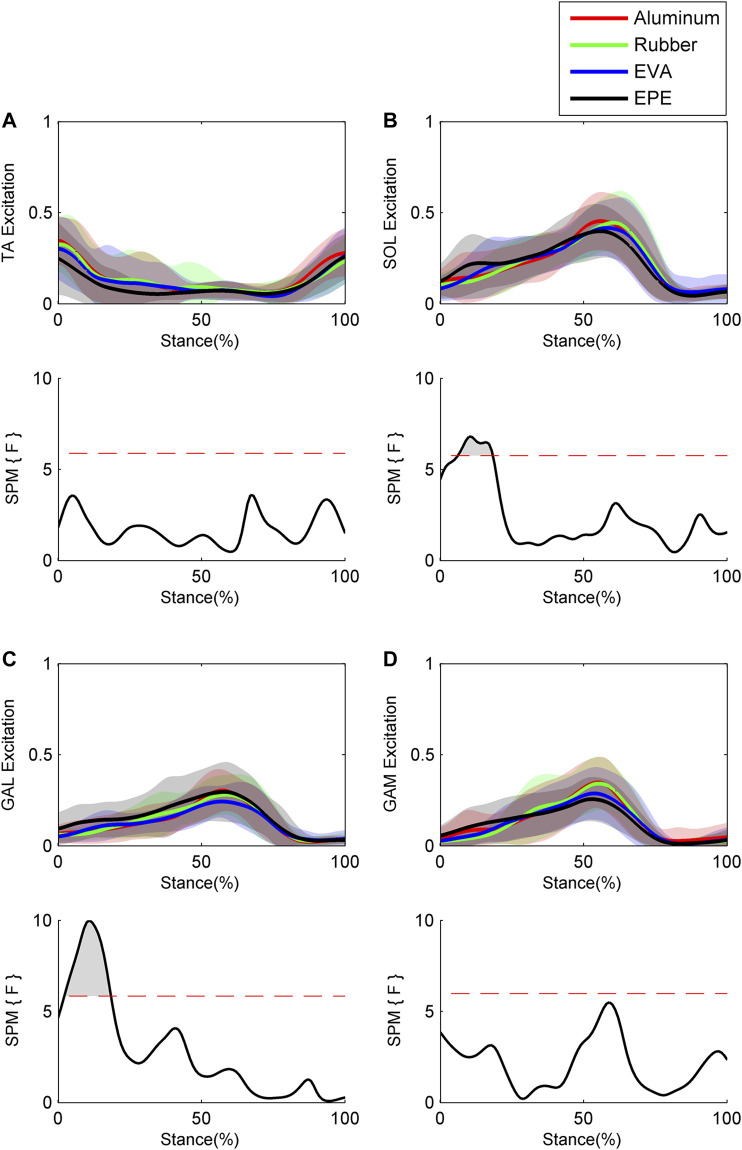
Ensemble average curves (continuous line) and standard deviation (shaded area) for muscle excitation during walking: **(A)** tibialis anterior (TA); **(B)** soleus (SOL); **(C)** gastrocnemius lateralis (GAL); and **(D)** gastrocnemius medialis (GAM). Data are reported for the stance phase of walking with 0% being heel strike and 100% being toe-off events. The shaded areas in the SPM figure indicate that a significant difference was found (*p* < 0.05) during this period.

Results of GRFs and muscle excitations are shown in Figure 4 and Figure 5, respectively. During the 10–19% of stance phase, vertical GRF on the most compliant surface EPE was larger than that on the other three surfaces (*p* < 0.001, effect size *f*: 0.26–0.34). During the 60–91% of stance phase, vertical GRF decreased as compliance of surfaces increased within the force plate, EVA, and EPE surfaces (*p* < 0.001, effect size *f*: 0.42–0.59). As for anterior-posterior GRF, significant difference existed during the 15–25% (*p* = 0.001, effect size *f*: 0.27–0.31) and 64–69% (*p* = 0.016, effect size *f*: 0.28–0.34) of stance phase. Anterior-posterior GRF on the EPE surface was larger than GRF on the other three surfaces during the 15–25% of stance phase. No obvious trend occurred in anterior–posterior GRF among surfaces during the 64–69% of stance phase. GRFs in both directions remained almost the same on the force plate and rubber surfaces during the stance phase. Excitations of SOL and GAL on EPE surface were significantly larger than other surfaces during the early stance phase (*p* = 0.006, effect size *f*: 0.18–0.27 and *p* = 0.001, effect size *f*: 0.24–0.38, respectively). Peak excitations of SOL and GAM tended to decrease as compliance of walking surfaces increased within the force plate, EVA, and EPE surfaces, although no significant difference existed (SOL: effect size *f* = 0.17 at 17% of the stance phase; GAM: effect size *f* = 0.29 at 59% of the stance phase).

## Discussion

In this study, subjects were required to walk on surfaces with four levels of compliance. Time-varying ankle moment and stiffness were estimated from GRFs, EMG, and three-dimensional reflective marker trajectories during the whole stance phase *via* an EMG-driven musculoskeletal model. Two main findings in our results were that 1) surface compliance affected the regulations of the ankle moment and stiffness and 2) the effect of the surfaces compliance on ankle moment and stiffness regulations varied in different subphases.

Surface compliance had different effects on ankle moment regulations during the early and mid-stance phases. During the early stance phase, the ankle dorsiflexion moment is a preparation for weight acceptance and provides a deceleration of the foot when landing (Gray and Basmajian, 1968; Hunt et al., 2001). Reduced dorsiflexion moments were observed when the heel stroke on surfaces with increased compliance due to the better cushioning property of these surfaces. During the mid-stance phase, larger ankle plantar flexion moments were generated on surfaces with increased compliance to prevent lower limb collapse, improving upper body support and stability (Winter, 1980; Kepple et al., 1997; Sadeghi et al., 2001). As almost no difference in the vertical and anterior–posterior GRFs among surfaces was found during this subphase, the larger ankle plantar flexion moment observed on surfaces with increased compliance might be due to larger moment arms. While walking on surfaces with increased compliance, the plantar center of pressure (COP) might advance more quickly to reach the full foot contact earlier in order to maintain stability (Zhang and Li, 2014). Thus, the distance between the COP and the ankle joint center enlarged, resulting in larger moment arm and ankle plantar flexion moment during the mid-stance phase. During the late stance phase, the ankle plantar flexion moment contributes to the forward acceleration (Kepple et al., 1997). Forward accelerations might be similar among surfaces as walking speeds were kept similar, which may explain why there was no difference in ankle moments during this subphase (Peterson et al., 2010; Peterson et al., 2011).

Ankle stiffness regulations in response to surface compliance were different between the mid- and late stance phases. During the mid-stance phase, the ankle stiffness increased with the increase in surfaces compliance, which was consistent with the leg stiffness regulations while running on compliant surfaces (Ferris et al., 1998). It has been shown that larger leg stiffness while running on compliant surfaces helped to keep the vertical location of the center of mass (COM) the same as that on rigid surfaces, allowing humans to maintain steady gait on different surfaces (Ferris et al., 1998; Ferris et al., 1999). Computer simulation showed that the leg stiffness depended on the joint stiffness of the lower limb, especially the ankle stiffness (Farley et al., 1998; Farley and Morgenroth, 1999). It should be noted that previous studies only calculated the average leg stiffness of the whole stance phase (Ferris et al., 1998; Ferris et al., 1999), while our study estimated the time-varying ankle stiffness during the whole stance phase and found that higher compliance of surfaces increased ankle stiffness during the mid-stance phase, but that decreased during the late stance phase. Our results showed that ankle stiffness increased only during the mid-stance phase on compliant surfaces, which may lead to efficient gait as increased ankle stiffness during the whole stance phase required higher energy cost (Moore et al., 2014; Li et al., 2021).

Previous studies have shown that alterations in ankle muscle excitations can change ankle stiffness (Trevino and Lee, 2018; Whitmore et al., 2019; Wind and Rouse, 2020). Our results showed that excitations of SOL and GAL on the surface with the lowest level of compliance were significantly larger than those on the other three surfaces during the early stance phase, which is consistent with that ankle plantar flexion muscle excitations increased following stepping on compliant surfaces (Marigold and Patla, 2005). As there was an electromechanical delay, the increased muscle excitations on compliant surfaces during the early stance phase might contribute to the larger ankle stiffness during the mid-stance phase (Lloyd and Besier, 2003). Our results also showed that during the late stance phase, peak excitations of SOL and GAM increased as compliance of walking surfaces decreased (no significant difference existed), while peak excitation of GAL showed no such trend. Increased peak excitations of SOL and GAM might contribute to the increase in peak ankle stiffness during the late stance phase as they are two of the major ankle plantar flexors (Wickiewicz et al., 1983; Silver et al., 1985).

The elastic modulus of materials reported in the Materials and Methods was obtained from the relationship of stress and strain (0–300 kPa), and this value was comparable between the EVA and EPE. It should be noticed that these two materials had a non-linear mechanical behavior. If we took only the part of the relationship of stress and strain into account, where stress is below 30 kPa, the obtained elastic modulus (0–30 kPa) of the EVA was more than twice of the EPE (0.25 and 0.10 MPa, respectively). Thus, the compliance of EVA and EPE was quite different during the initial contact with surfaces, probably leading to the difference in ankle moment and stiffness between two surfaces.

Alterations in the ankle moment and stiffness were found in the rubber, EVA, and EPE surfaces, and it is notable that both ankle moments and ankle stiffness remained almost the same while walking on the force plate and rubber surfaces. This might be associated with the mechanical properties of human tissues. The elastic modulus of plantar tissue is about 0.7 MPa (Ledoux and Blevins, 2007), while this value of the EVA and EPE was 0.34 and 0.29 MPa, respectively. The EVA and EPE are softer than plantar tissue, and the deformation during gait mainly occurred on the walking surfaces. The elastic modulus of the force plate and rubber was about 70000 and 4.10 MPa, respectively, which was significantly larger than the plantar tissue. Hence, the deformation during gait primarily occurred on the plantar tissue while walking on them. As the vertical GRF remained almost the same on the force plate and rubber surfaces during the whole stance phase, similar deformation occurred on plantar tissue, leading to similar ankle moment and stiffness regulations (Ferris et al., 1999). There are some limitations in this study. The tested order of different surfaces was not randomized, which may have an influence on the results. However, subjects were given time to familiarize with the tested surface before recording and took a 15-min rest between two surface conditions. This adapting practice and rest between two conditions can minimize the effect of previous walking trials on other surfaces. The effect sizes *f* of differences in the ankle moment and stiffness were considered medium in this study (Cohen, 1969), which is lower than the calculated value of the pilot study. As such, the results might be considered as exploratory. Step length and stride frequency were not strictly controlled in our experiment, which may have an effect on the values of GRFs and ankle moments (Allet et al., 2011). Only four superficial muscles were monitored and taken into account in the model to calculate ankle stiffness, leading to lower ankle stiffness estimates as the contributions of deeper muscles were neglected.

The ankle joint plays a key role in adjusting leg mechanics to adapt to alterations in surface properties (Ferris et al., 1998; Zanetti et al., 2013; Kessler et al., 2020). Our findings about ankle mechanical adaptations could be instructive to the sport surface design (Zanetti et al., 2013). Joint stiffness is one of the key parameters to develop passive multibody models for human body simulations (Pascoletti et al., 2019, 2020). Our findings provided ankle stiffness information for the construction of human simulation models on surfaces with different compliance.

## Conclusion

Our study provides insights into how humans regulate ankle moment and stiffness during the whole stance phase while walking on surfaces with different levels of compliance. Surfaces with higher levels of compliance increased the ankle plantar flexion moment and stiffness during the mid-stance phase, while decreased the ankle stiffness during the late stance phase. The ankle moment and stiffness regulations in response to surface compliance primarily helped to prevent lower limb collapse and improve stability on surfaces with different compliance. Our work gave a comprehensive understanding about the regulations of ankle biomechanics including ankle moment and ankle stiffness and might be instructive to sports surfaces design and passive multibody model development.

## Data Availability

The raw data supporting the conclusions of this article will be made available by the authors, without undue reservation.

## Supplementary Material

Supplementary Figure 1 Stress–strain curves of three materials.

Supplementary Figure 2 Marker placements on body of subjects, modified based on the marker set of gait2392 model in OpenSim.
