# Supplementary figures and images for: How Compliance of Surfaces Affects Ankle Moment and Stiffness Regulation During Walking
Source: Front Bioeng Biotechnol. 2021 Oct 5;9:726051. doi: 10.3389/fbioe.2021.726051 (PMC8523823; doi:10.3389/fbioe.2021.726051)

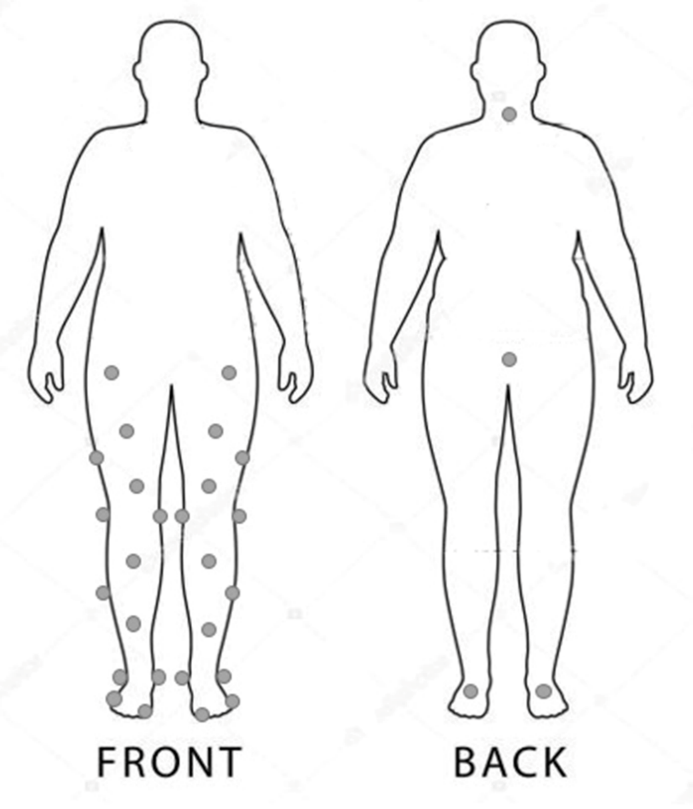

Supplement: Supplementary file 1 [file image2.tif]

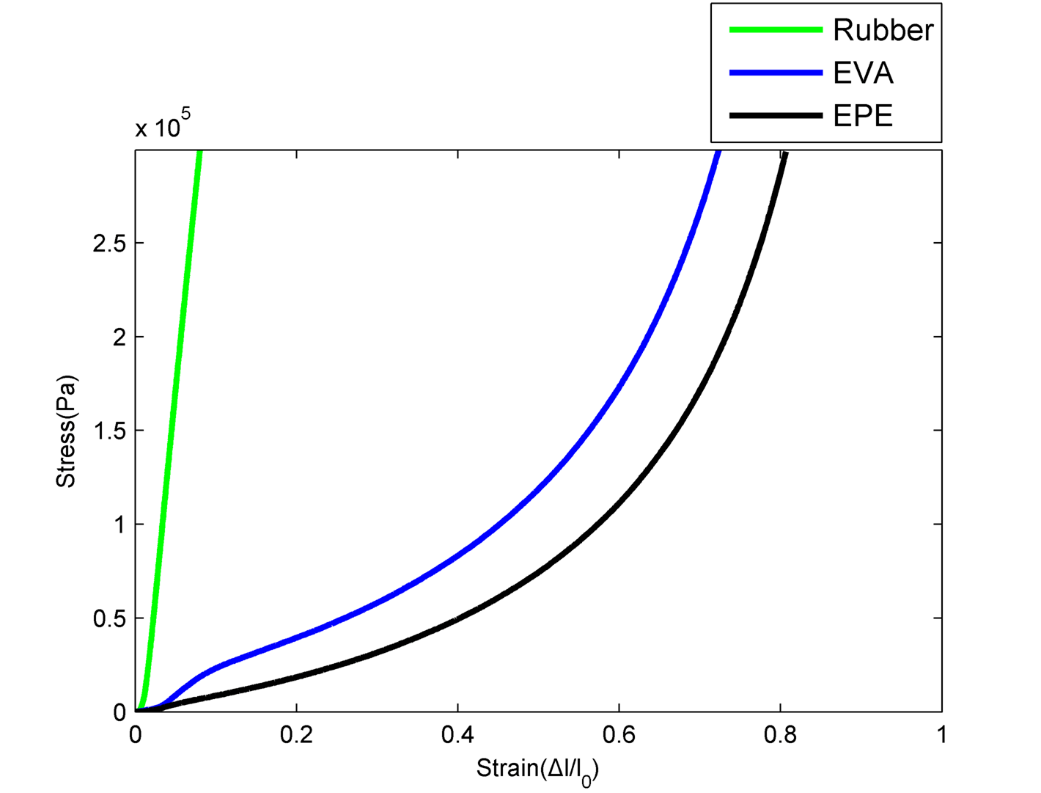

Supplement: Supplementary file 2 [file image1.tif]
